# Supplementary material for: Differential regulation of the eicosanoid biosynthesis pathway in response to Enterocytozoon hepatopenaei infection in Litopenaeus vannamei
Source: PLoS One. 2025 Oct 17;20(10):e0334906. doi: 10.1371/journal.pone.0334906 (PMC12533846; doi:10.1371/journal.pone.0334906)
Supplement: S1 Table — (DOCX) [file pone.0334906.s001.docx]

**S1 Table** Primer sequences and PCR conditions for the quantitative real-time PCR analysis in *L. vannamei*

| Genes | Abbr. | Primer sequences | Tm (°C) | Product size (bp) | R^2^ | E |  |  |  |  |  |  |
| --- | --- | --- | --- | --- | --- | --- | --- | --- | --- | --- | --- | --- |
| *Cytosolic phospholipase A2* | *LvcPLA2* | F: 5’ CTCTCGAGCCGGAAGAGCAG 3’ | 62 | 240 | 1.000 | 95.1 |  |  |  |  |  |  |
|  |  | R: 5’ TGTGGCCTCAAGAGCAGTGG 3’ |  |  |  |  |  |  |  |  |  |  |
| *Cyclooxygenase* | *LvCOX* | F: 5’ GGGGTCAAAGGCCCCAAGGAGTACC 3’ | 62 | 305 | 0.999 | 95.7 |  |  |  |  |  |  |
|  |  | R: 5’ AGGTAAGGTGGGAAATCCTCGCCGTTG 3’ |  |  |  |  |  |  |  |  |  |  |
| *Prostaglandin F synthase* | *LvPGFS* | F: 5’ GGAGAAGTAATGCAGGCTGT 3’  R: 5’ GCCAGGTCTCAATGTAATCC 3’ | 60 | 352 | 0.991 | 102.4 |  |  |  |  |  |  |
| *Elongation factor 1α* | *LvEF1α* | F: 5’ ATGGGCTGGTGGAAGAAGC 3' | 63 | 131 | 0.992 | 97.7 |  |  |  |  |  |  |
|  |  | R: 5’ TGAAGGGGAAGACGGAGGG 3' |  |  |  |  |  |  |  |  |  |  |
|  |  |  |  |  |  |  |  |  |  |  |  |  |
|  |  |  |  |  |  |  |  |  |  |  |  |  |
|  | | | | | | |  |  |  |  |  |  |
|  |  |  |  |  |  |  |  |  |  |  |  |  |
|  |  |  |  |  |  |  |  |  |  |  |  |  |
|  |  |  |  |  |  |  |  |  |  |  |  |  |
|  |  |  |  |  |  |  |  |  |  |  |  |  |
|  |  |  |  |  |  |  |  |  |  |  |  |  |
|  |  |  |  |  |  |  |  |  |  |  |  |  |
|  |  |  |  |  |  |  |  |  |  |  |  |  |
|  |  |  |  |  |  |  |  |  |  |  |  |  |
|  |  |  |  |  |  |  |  |  |  |  |  |  |
|  |  |  |  |  |  |  |  |  |  |  |  |  |
|  |  |  |  |  |  |  |  |  |  |  |  |  |
