# Supplementary material for: Differential regulation of the eicosanoid biosynthesis pathway in response to Enterocytozoon hepatopenaei infection in Litopenaeus vannamei
Source: PLoS One. 2025 Oct 17;20(10):e0334906. doi: 10.1371/journal.pone.0334906 (PMC12533846; doi:10.1371/journal.pone.0334906)

A

```
Human(NP_000953.2)vs L. vannamei Sequence (XP_027218437.1)identities 101/200

The epitope used to raise antibody against human COX is highlighted in yellow

XP_027218437.1      MSTSVKSMATTGQRGDKGGGRVVFGVAGVILAASFLFMRTPVHETPAPTPTAAVIDYDPC     60
NP_000953.2         -----------------------MSRSLLLWFLLFLLLLPPLPVL--LADPGAPTPVNPC     35
                                            :. : ::    **::  *:      :  .*    :**

XP_027218437.1      CAYPCENQGICMSKPDRNYTCDCTGTGYYGTNCEIPTWSTAIKTKLKPDPEWLHTFITSH     120
NP_000953.2         CYYPCQHQGICVRFGLDRYQCDCTRTGYSGPNCTIPGLWTWLRNSLRPSPSFTHFLLTHG     95
                    * ***::****:     .* **** *** * ** **   * ::..*:*.*.: * ::*

XP_027218437.1      SWLWAIINRIPFIHKRLMTYVYLSRGDLVDSPPTYESEHSYITLNAYYNESFYARTLPPV     180
NP_000953.2         RWFWEFVNA-TFIREMLMRLVLTVRSNLIPSPPTYNSAHDYISWESFSNVSYYTRILPSV     154
                     *:* ::*   **:: **  *   *.:*: *****:* *.**: ::: * *:*:* ** *

XP_027218437.1      PEHCPTPMGVKGPKEYPDVDELIKKVFLRREFIPEPHDTNVLFQYYAQHFTHQFFRTDYK     240
NP_000953.2         PKDCPTPMGTKGKKQLPDAQLLARRFLLRRKFIPDPQGTNLMFAFFAQHFTHQFFKTSGK     214
                    *:.******.** *: **.: * ::.:***:***:*:.**::* ::*********:*. *

XP_027218437.1      KGPHLTKGTG-GVDVSNIYGLTEQDRRALRSGVDGKLRTQKINGEDFPPYLKDVPGISMD     299
NP_000953.2         MGPGFTKALGHGVDLGHIYGDNLERQYQLRLFKDGKLKYQVLDGEMYPPSVEEAPV-LMH     273
                     ** :**. * ***:.:*** . : :  **   ****: * ::** :** :::.*   *.

XP_027218437.1      YPPHIPIPEEGKFALGHPFFALLPGLFAYATIWVREHNRVCDELVKVHPDWDDERIYQTA     359
NP_000953.2         YPRGI--PPQSQMAVGQEVFGLLPGLMLYATLWLREHNRVCDLLKAEHPTWGDEQLFQTT     331
                    **  *  * :.::*:*: .*.*****: ***:*:******** *   ** *.**:::**:

XP_027218437.1      RLIIIGEVIKITIEDYVQHLSQYNLRLTFEPELTHGTRFQYHNRIHAEFNHLYHWHPLIP     419
NP_000953.2         RLILIGETIKIVIEEYVQQLSGYFLQLKFDPELLFGVQFQYRNRIAMEFNHLYHWHPLMP     391
                    ***:***.***.**:***:** * *:*.*:***  *.:***:***  ***********:*

XP_027218437.1      DTLNVSGTDYAIMDMAYSTAPVFKHGLDEFIHSMVNSRAGALT-SRNHAHALYPVLKKVI     478
NP_000953.2         DSFKVGSQEYSYEQFLFNTSMLVDYGVEALVDAFSRQIAGRIGGGRNMDHHILHVAVDVI     451
                    *:::*.. :*:  :: :.*: :..:*:: ::.:: .. ** :  .**  * :  *  .**

XP_027218437.1      ENGRELRFQGVNAYRRRFGMVPFTSFEDMTGETELAAILEEMYGDIEAVEYYVGLLAERP     538
NP_000953.2         RESREMRLQPFNEYRKRFGMKPYTSFQELVGEKEMAAELEELYGDIDALEFYPGLLLEKC     511
                    .:.**:*:* .* **:**** *:***:::.**.*:** ***:****:*:*:* *** *:

XP_027218437.1      GP-SITPLTMVNIGGPWSVKGLLANPICSPRYWKPSTFGGEEGFNIIKTASLEKLFCLNM     597
NP_000953.2         HPNSIFGESMIEIGAPFSLKGLLGNPICSPEYWKPSTFGGEVGFNIVKTATLKKLVCLNT     571
                     * **   :*::**.*:*:*****.******.********** ****:***:*:**.***

XP_027218437.1      KGSCESIGFKVPQGTP-------------        613
NP_000953.2         K-TCPYVSFRVPDASQDDGPAVERPSTEL        599
                    * :*   :.*:**:.:
```

Highlighted in yellow (epitope), NP_000953.2: LSGYFLQLKFDPELLFGVQFQYRNRIAMEFNHLYHWHPLMP / DSFKVGSQEYSYEQFLFNTSMLVDYGVEALVDAFSRQIAGRIGGGRNMDHHILHVAVDVI / RESREMRLQPFNEYRKRFGMKPYTSFQELVGEKEMAAELEELYGDIDALEFYPGLLLEKC / HPNSIFGESMIEIGAPFSLKGLLGNPICSPEYWKPSTFG

- Predicted LvCOX protein size = 69.4 kDa + 2 glycosylation x 2.5 kDa resulted in the predicted LvCOX at 74 kDa

B

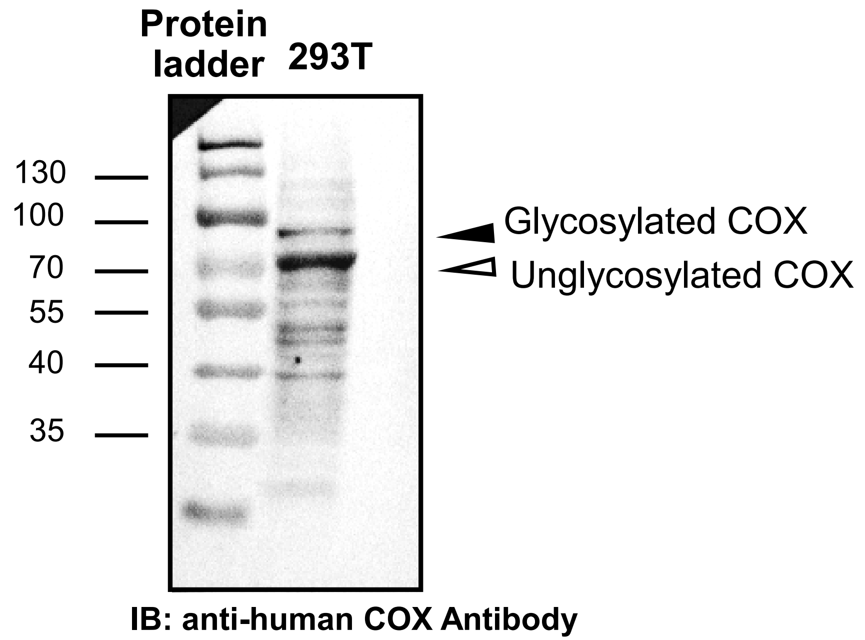

C

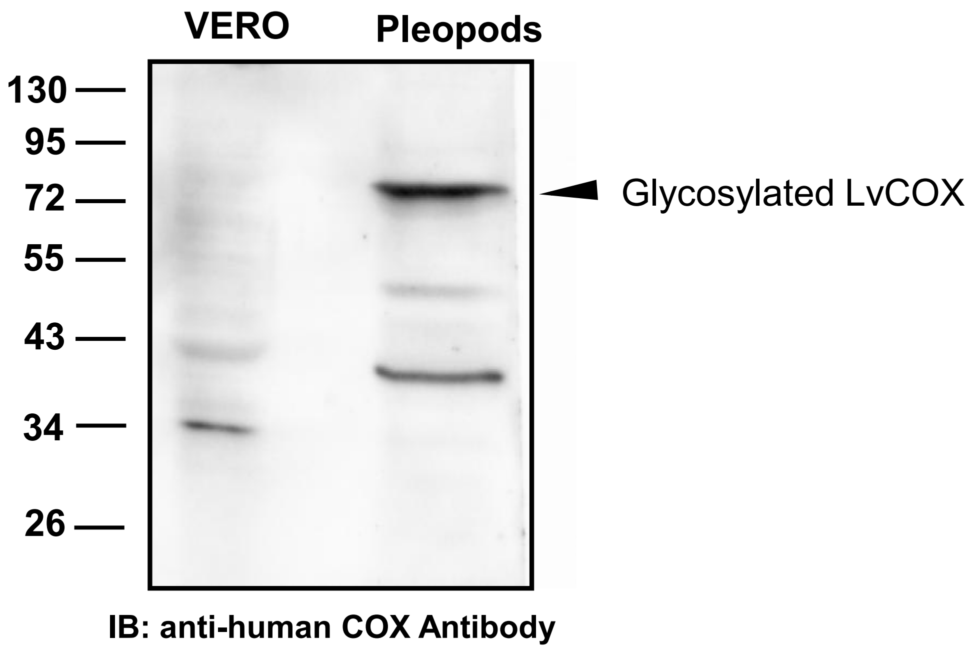

A

L. vannamei sequence used to raise polyclonal antibody

>ROT80223.1 prostaglandin F synthase [*Penaeus vannamei*]

```
MAAQVPKLALNNGRNIPILGLGTWKSKPGEVTQAVKDAIACGYRHIDCALAYSNEAEVGA
AIKAKIEDGTVKREDLFITSKLWNTFHSRPLVTASLKQSLANLGLDYLDLYLIHWPMGYQ
ENAALFPKDENGKFIYTDVDYLETWSGMEDAVDQGLSKSIGISNFNSEQIQRILGNCRIK
PANHQIECHPYLNQKKLIDFCHKNGITVTAYSPLGSPDRPWAKPGEPQLMDDTRIISIAE
KYKKSPAQVLIRYQIQRQVIVIPKSVTKARIEANFQVLDFTLSDEDIKVIDSFDCNGRLL
HLDWVKDHKYWPFNIEF
```

- Predicted LvPGFS protein size = 35.89 kDa
- PGFS is also known for being glycosylated, which would increase the protein size slightly.

B

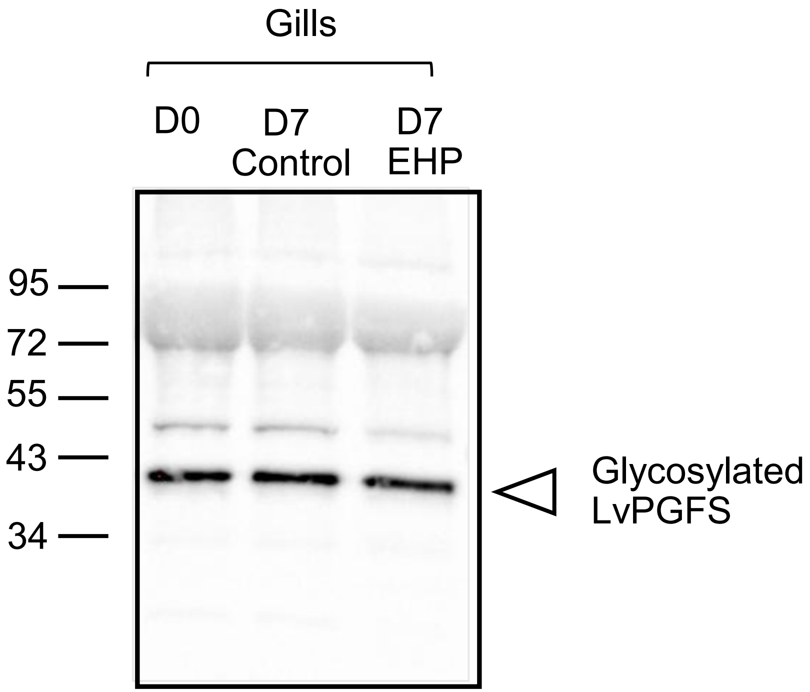

Supplement: S4 Data — (PDF) [file pone.0334906.s005.pdf]
