# Supplementary material for: Differential regulation of the eicosanoid biosynthesis pathway in response to Enterocytozoon hepatopenaei infection in Litopenaeus vannamei
Source: PLoS One. 2025 Oct 17;20(10):e0334906. doi: 10.1371/journal.pone.0334906 (PMC12533846; doi:10.1371/journal.pone.0334906)
Supplement: Attachment — Submitted filename: Response to Reviewers_Rebuttal_R2.docx [file pone.0334906.s009.docx]

National Center for Genetic Engineering and Biotechnology

National Science and Technology Development Agency

Phahonyothin Road, Khlong Nueng, Khlong Luang

Pathum Thani, Thailand

15 September 2025

Dear Editor and Reviewers,

We are submitting a revised manuscript titled “**Differential regulation of the eicosanoid biosynthesis pathway in response to *Enterocytozoon hepatopenaei* infection in *Litopenaeus vannamei***” **(PONE-D-25-23707_R2)** to be considered for research article publication in **PLOS One**. We have modified the manuscript based on the reviewers’ comments. The point-by-point responses are provided below.

---

We've checked your submission and before we can proceed, we need you to address the following issues:

1. To comply with PLOS ONE submissions requirements, in your Methods section, please provide additional information regarding the experiments involving animals and ensure you have included details on methods of anesthesia and/or analgesia.

**Answer:** The information regarding anesthesia was previously in the materials and methods section under the heading of EHP cohabitation experiment. However, for clarity, we have made a separate heading for “Methods of anesthesia and/or analgesia”

Reviewer's Responses to Questions

1. If the authors have adequately addressed your comments raised in a previous round of review and you feel that this manuscript is now acceptable for publication, you may indicate that here to bypass the “Comments to the Author” section, enter your conflict of interest statement in the “Confidential to Editor” section, and submit your "Accept" recommendation.

Reviewer #1: All comments have been addressed

Reviewer #2: All comments have been addressed

Reviewer #3: (No Response)

**Answer:** The authors thanked all the reviewers for their comments.

2. Is the manuscript technically sound, and do the data support the conclusions?

The manuscript must describe a technically sound piece of scientific research with data that supports the conclusions. Experiments must have been conducted rigorously, with appropriate controls, replication, and sample sizes. The conclusions must be drawn appropriately based on the data presented.

Reviewer #1: Yes

Reviewer #2: Yes

Reviewer #3: Partly

**Answer:** The authors understand that the difference in the levels of eicosanoids between samples collected on day 0, 7, and 21 from the control groups was not explained to the satisfaction of Reviewer #3. We added a paragraph in the discussion section (line 594-614) with references to try to convey our findings.

3. Has the statistical analysis been performed appropriately and rigorously?

Reviewer #1: Yes

Reviewer #2: Yes

Reviewer #3: Yes

**Answer:** We thanked the reviewers for their comments.

4. Have the authors made all data underlying the findings in their manuscript fully available?

The PLOS Data policy requires authors to make all data underlying the findings described in their manuscript fully available without restriction, with rare exception (please refer to the Data Availability Statement in the manuscript PDF file). The data should be provided as part of the manuscript or its supporting information, or deposited to a public repository. For example, in addition to summary statistics, the data points behind means, medians and variance measures should be available. If there are restrictions on publicly sharing data—e.g. participant privacy or use of data from a third party—those must be specified.

Reviewer #1: Yes

Reviewer #2: Yes

Reviewer #3: No

**Answer:** The authors have provided all the raw data for the experiment in the Supplementary data section. In this revision, we have added the statistical analysis of eicosanoids in S2 Data file in the Supplementary section and the statistical analysis of qPCR is also included in S5 Data.

5. Is the manuscript presented in an intelligible fashion and written in standard English?

PLOS ONE does not copyedit accepted manuscripts, so the language in submitted articles must be clear, correct, and unambiguous. Any typographical or grammatical errors should be corrected at revision, so please note any specific errors here.

Reviewer #1: Yes

Reviewer #2: Yes

Reviewer #3: Yes

**Answer:** We thanked the reviewers for their comments

6. Review Comments to the Author

Please use the space provided to explain your answers to the questions above. You may also include additional comments for the author, including concerns about dual publication, research ethics, or publication ethics. (Please upload your review as an attachment if it exceeds 20,000 characters)

**Reviewer #1:** All comments have been addressed. Therefore, I recommend it for accepting in your esteemed journal.

**Answer:** We thanked Reviewer#1 for his/her comments.

**Reviewer #2**: The revised manuscript shows substantial improvement over the original submission. The authors have provided additional methodological details, clarified data interpretation, and strengthened the connection between EHP infection, tissue-specific responses, and eicosanoid regulation. The study is scientifically rigorous, and the revisions address most concerns raised previously. The manuscript is now close to being suitable for publication, with only minor issues requiring attention.

1. Terminology: Ensure consistent terminology for infection stages (e.g., “early infection” vs. “day 7” and “late infection” vs. “day 21”). At times both are used interchangeably within a single paragraph, which may confuse readers.

**Answer**: Thank you for your suggestion. We have adjusted the terminology to only Day 7 and Day 21 based on the reviewer’s recommendation. The change can be found throughout the manuscript.

2. Grammar and style: A few sentences remain overly long and complex, particularly in the Introduction. Breaking these into shorter, more direct statements would improve readability.

**Answer**: Thank you for your suggestion. We apologize for the long sentence. We have tried to cut down the sentence in the abstract, introduction, and discussion section of the manuscript based on the reviewer’s suggestions.

**Reviewer #3:** The authors responded to the comments by suggesting that the potential influence of uninfected shrimp was mostly determined by other factors such as shrimp age and rearing environment. Please provide references and include them in the section of discussion.

**Answer**: We added a paragraph in the discussion section (line 594-614) with references to try to convey our findings. Although the aging process and rearing environments can impact the levels of eicosanoids in the organisms, these are studies that were performed in mammals. Therefore, we have adjusted the reference to explain our findings based on the accumulation of nutrients, the molting period, and the stress caused by water temperature to suit the reference that we obtained in crustaceans.

We thank both the academic editor and reviewers for their invaluable comments that help improve the quality of our manuscript. We are looking forward to hearing a positive response from you.

Wananit Wimuttisuk

[wananit.wim@biotec.or.th](mailto:wananit.wim@biotec.or.th)

National Science and Technology Development Agency, Thailand
